# Seasonal changes in sleep duration and sleep problems: A prospective study in Japanese community residents

**DOI:** 10.1371/journal.pone.0215345

**Published:** 2019-04-18

**Authors:** Masahiro Suzuki, Tetsuya Taniguchi, Ryuji Furihata, Katsushi Yoshita, Yusuke Arai, Nobuo Yoshiike, Makoto Uchiyama

**Affiliations:** 1 Department of Psychiatry, Nihon University School of Medicine, Tokyo, Japan; 2 Division of Mathematics, Department of Liberal Education, Nihon University School of Medicine, Tokyo, Japan; 3 Department of Food and Human Health Science, Osaka City University, Graduate School of Human Life Science, Osaka, Japan; 4 Department of Nutrition, Faculty of Health Care Sciences, Chiba Prefectural University of Health Sciences, Chiba, Japan; 5 Department of Health and Welfare Public Policy, Aomori University of Health and Welfare, Graduate School of Health Sciences, Aomori, Japan; Neurocenter of Southern Switzerland, SWITZERLAND

## Abstract

**Background:**

A scientific understanding of the effects of seasonal changes on sleep duration and sleep problems such as insomnia and hypersomnia has yet to be elucidated; however, such an understanding could aid the establishment of an optimal sleep hygiene program to treat such problems.

**Methods:**

We investigated the effects of seasonal changes on sleep duration and sleep problems in Japanese community residents. Data on 1,388 individuals aged 15–89 years who participated in the Survey of Seasonal Variations in Food Intakes conducted by the National Institute of Health and Nutrition of Japan (2004–2007) were analyzed. Participants completed a questionnaire including items on sleep duration and sleep problems (difficulty initiating sleep [DIS], difficulty maintaining sleep [DMS]/early morning awakening [EMA], and excessive daytime sleepiness [EDS]). Data were prospectively collected at four time points (spring, summer, fall, and winter).

**Results:**

Seasonal changes in sleep duration were found, with the longest in winter and the shortest in summer (winter–summer difference: 0.19 h). The seasonality of sleep duration was influenced by age, sex, and residential area. In terms of age, seasonal changes in sleep duration were found in the middle and old age groups, but not in the young age group. Seasonal changes in the frequencies of sleep problems were found for some items in the young age group (DMS/EMA and EDS) and middle age group (DIS and DMS/EMA); however, no such changes were observed in the old age group.

**Conclusion:**

Seasonal effects on sleep and sleep problems were found in Japanese community residents, but these varied between age groups. Furthermore, seasonal changes in sleep duration were influenced by sex and residential area.

## Introduction

Sleep is essential for recovery and restoration of the body and brain in humans, and a lack of sleep is associated with poor physical and mental performance [1]. Over the last few decades, there has been growing evidence to suggest that not only short, but also long sleep is associated with adverse health outcomes, including type 2 diabetes [2], hypertension [3], cardiovascular disease [4], obesity [5], respiratory disorders [6], depression [7], and total mortality [8, 9]. Accordingly, a wide range of studies on sleep and sleep disorders has been actively carried out with respect to a variety of health statuses.

Seasonal changes in behavior and physiology have been recognized since ancient times [10]. Especially, seasonal variation in sleep, as well as in mood, has been described in many studies. However, although it could help establish the optimal sleep hygiene program for sleep problems such as insomnia or hypersomnia and could be expected to improve sleep-related public health, a scientific understanding of the effects of seasonality on sleep and sleep problems has yet to be elucidated.

Seasonal changes in sleep duration have mostly been studied in relation to seasonal affective disorder (SAD), which is a type of depression that has a seasonal pattern, typically occurring in the fall and winter with remission in the spring or summer [11]. In clinical settings, patients with winter-type SAD have been reported to have longer sleep duration and more severe depressive symptoms in winter than in summer [11]. To a lesser degree, studies applying the Seasonal Pattern Assessment Questionnaire (SPAQ), a self-report questionnaire that retrospectively assesses the magnitude of seasonal changes in mood and behavior, have documented prolongation and reduction of sleep duration in winter and summer among those not suffering from SAD [12–14]. A telephone survey of Maryland residents reported that the average sleep duration during winter was 7.41 h, compared with 7.05 h during summer [13]. Volkov et al. reported that college students in the US showed 24.6 min longer sleep duration in winter compared with summer [14].

Seasonal changes in sleep problems have been studied mainly in Northern European countries to investigate the association with the midnight sun and the dark period. Epidemiological studies conducted in these countries have reported an effect of seasonality on sleep quality, predominately with poorer sleep during the winter months [15–17], although a negative finding was also reported [18]. In a study investigating month-to-month variation in sleeping problems in six regions in Northern and Central Asia and Alaska, sleeping problems were linked to certain seasonal photic and temperature extremes [19]. In contrast to these reports on mainly high latitude areas, a recent epidemiological study conducted in Japan, which is in the temperate zone, found no association between insomnia and seasonality [20].

Although important findings on seasonal changes in human sleep have been reported, there may be an inevitable limitation of recall bias because in most of these prior studies, the SPAQ was used. Only a prospective study that investigated the seasonality of sleep and sleep-related problems three times across different seasons (spring, fall, and winter) has been conducted in the Arctic circle [21]. However, seasonal changes in sleep duration and sleep problems in the temperate zone have not been clarified in a prospective study. Moreover, no studies have investigated accurate changes of sleep across four seasons in a prospective manner.

In the present study, to assess how sleep duration and the frequencies of sleep problems change across the seasons in temperate regions, we prospectively surveyed these items at four time points (spring, summer, fall, and winter) in Japanese community residents. With aging, the daily amount of sleep decreases and the incidence of sleep disturbance increases [22]. Therefore, the seasonal patterns of sleep duration and sleep problems could differ by age group; however, this has not been studied in populations comprising a wide range of ages. In the present study, we also investigated age group differences on the seasonality of sleep duration and sleep problems.

## Methods

### Participants

This study was conducted based on data derived from the Survey of Seasonal Variations in Food Intakes conducted by the National Institute of Health and Nutrition of Japan between 2004 and 2007 [23]. This survey was carried out to provide fundamental insights regarding seasonal variation in regard to the National Health and Nutrition Survey, which has been conducted annually in autumn for over 60 years to obtain basic data for implementing effective measures for health promotion in Japan [24].

The primary aim of the present validation survey was to assess seasonal variation in food intake among Japanese community residents by collecting detailed dietary intake data at four time points (spring, summer, fall, and winter) using a method similar to that employed in a previous series of the National Health and Nutrition Survey [25]. For this purpose, 21 districts in the following 19 prefectures throughout Japan were selected as survey areas: Aomori, Iwate, Akita, Yamagata, Niigata, Chiba, Tokyo, Kanagawa, Ibaraki, Gunma, Nagano, Aichi, Okayama, Tokushima, Ehime, Kochi, Fukuoka, and Miyazaki. Since it was necessary to obtain eligible participants who were cooperative and had the skills necessary to complete detailed 3-day dietary records using food weighing methods four times a year, we employed the convenient sampling method. Specifically, the research collaborators (local health centers, local dietetic associations, or researchers in universities) at each survey site selected 25 to 30 households in which the main participants (mostly engaged in the household cooking) were expected to complete dietary records, with some consideration that these survey samples might not be too biased compared with the general population.

The present survey was conducted between May 2004 and March 2005 for seven districts, between May 2005 and March 2006 for eight districts, and between May 2006 and March 2007 for six districts. The survey districts in each year were selected from three areas in Japan: the north, middle, and south (the details of the categorization are mentioned later). For each year, the data were collected four times from the same households as follows: in May and June (spring), August and September (summer), November and December (fall), and February and March (winter). After collection, all data were gathered and sorted by season for analysis.

Data on sleep and sleep problems were collected using a questionnaire. Interviewers at each survey site trained in conducting dietary surveys visited selected households during each season and explained to the participants how to record dietary data. At the same time, they distributed the questionnaires, which included items on sleep and sleep problems, and instructed the households on how to complete them. The dietary data and completed questionnaires were collected by the interviewers during each season. The target population in the present study were individuals aged 15–89 years living in the selected households.

This survey was conducted in accordance with the Declaration of Helsinki and was approved by the ethics committee of the National Institute of Health and Nutrition, Tokyo, Japan. Sufficient explanations were given orally and by visual materials to all households from which data were collected. All participants provided written informed consent for collaboration in this survey.

### Measures

The self-administered questionnaires were composed of 19 items concerning (1) consciousness about diet and nutrition, (2) exercise habits, (3) food preferences, (4) sleep and daytime sleepiness, (4) smoking, and (5) alcohol drinking. The questionnaires were delivered to family members aged 15 years and over by the main participants in the selected households and were required to be filled out by each family member.

The following questions about sleep and daytime sleepiness during the past 2 weeks were embedded in the questionnaire:

1. “Approximately how many hours of actual sleep per night did you get during the past two weeks? (The time you spent asleep may differ from the time you spent in bed, and does not include naps.)”: sleep duration
2. “During the past two weeks, how often have you had difficulty sleeping because of the following reasons?”
2–1. “I was unable to fall asleep within 30 minutes of getting into bed.” (not at all/less than once a week/once or twice a week/three or more times a week): difficulty initiating sleep (DIS)
2–2. “I woke up during the night or too early in the morning.” (not at all/less than once a week/once or twice a week/three or more times a week): difficulty maintaining sleep (DMS)/early morning awakening (EMA)
3. “During the past two weeks, have you ever had trouble staying awake when you were performing a task where you should never fall asleep, such as driving, eating, or engaging in social activities?” (not at all/less than once a week/once or twice a week/three or more times a week): excessive daytime sleepiness (EDS)

To evaluate the prevalence of sleep problems and daytime sleepiness, dichotomized variables were also created for DIS, DMS/EMA, and EDS. Participants who selected “three or more times a week” for questions 2–1 and 2–2 were defined as having DIS and DMS/EMA, respectively. Participants who selected “once or twice a week” or “three or more times a week” for question 3 were defined as having EDS.

### Demographic variables

Variables analyzed included age, sex, and residential area. Age was divided into three groups by 25-year intervals: young (15–39), middle (40–64), and old (65–89). The districts were categorized into three areas according to latitude: north (five districts lying between 37.4°N and 40.9°N), middle (eight districts lying between 35.2°N and 36.3°N), and south (eight districts lying between 31.9°N and 34.4°N).

### Statistical analysis

Seasonal changes in sleep duration, as well as the effects of age, sex, and residential area, were assessed using a linear mixed model based on the restricted maximum likelihood approach, with sleep duration as the dependent variable and age group (*age*; young, middle, and old), sex (*sex*; male and female), and residential area (*area*; north, middle, and south) as the fixed factors. Season (*season*; spring, summer, fall, and winter) was treated as the repeated-measures factor. Initially, we tested the four-way interaction (*season* × *age* × *sex* × *area*) using the full model including all main effects and two-, three-, and four-way interactions. When the four-way interaction was not significant, we removed it and re-estimated the model. We then evaluated the three-way interactions. Next, we removed all nonsignificant three-way interactions and re-estimated the model. This procedure was then applied to the two-way interactions and, finally, to the main effects. Based on the final model, we assessed the seasonal changes in sleep duration, as well as their interactions with the other factors. In the mixed-model procedure, two models were considered: the first was the traditional homogeneous unstructured covariance model, and the second was a heterogeneous unstructured covariance model, which has a covariance for each age group. When both models converged, the likelihood ratio test was used to select the best fitted model. Significance was tested using Type II sum of squares, and the Kenward–Roger method was applied for estimating the denominator degrees of freedom. Estimated marginal means were calculated using the LSMEANS statement in PROC MIXED [26]. Estimated marginal means are predicted means that are calculated from the fitted model and are adjusted appropriately for any other variable. The p-value was adjusted by the Tukey–Kramer procedure. Seasonal changes in the frequencies (4-point score) of DIS, DMS/EMA, and EDS symptoms were analyzed using the Friedman test. This test was performed for the total sample as well as for each age group. Cochran's Q test was used to assess seasonal differences in the prevalence of sleep problems (DIS, DMS/EMA, and EDS). When a significant difference was found, post-hoc pairwise comparisons were performed using Bonferroni correction.

Given that adolescents differ from adults in terms of brain maturation, sleep homeostatic and circadian system function, and psychosocial milieu [27–29], we also performed a subgroup analysis for the young age group. For this analysis, the participants in the young age group were subdivided into adolescents (15–20 years) and young adults (21–39 years). Seasonal changes in sleep duration in these sub-age groups were assessed using the same mixed-model approach described above, but age group was replaced by the four-group categorization (adolescents, young adults, middle, and old). Seasonal changes in the frequencies (4-point score) of DIS, DMS/EMA, and EDS symptoms in each sub-age group were analyzed using the Friedman test. Cochran's Q test was used to assess seasonal differences in the prevalence of sleep problems (DIS, DMS/EMA, and EDS). When a significant difference was found, post-hoc pairwise comparisons were performed using Bonferroni correction.

Statistical significance was set at p<0.05. The mixed-model analyses were performed using SAS version 9.4 (SAS Institute Inc., Cary, NC, USA). The other analyses were conducted using SPSS version 24.0 (IBM Corporation, Armonk, NY, USA).

## Results

Of 1,568 participants who responded to the questionnaire at all four survey points, 180 were excluded because of missing data on one or more of the variables in the analysis (i.e., sex, sleep duration, DIS, DMS/EMA, or EDS). Therefore, the final sample used in the analyses comprised 1,388 participants. The participants’ background characteristics are shown in Table 1.

**Table 1:** Characteristics of the study participants (n = 1,388).

Age in years, n (%)	
Mean±SD	52.3±17.2
Range	
15–39	339 (24.4)
40–64	709 (51.1)
65–89	340 (24.5)
**Sex, n (%)**	
M	627 (45.2)
F	761 (54.8)
**Area, n (%)**	
North	308 (22.2)
Middle	558 (40.2)
South	522 (37.6)

Abbreviations: SD = standard deviation, M = male, F = female.

### Seasonal changes in sleep duration and the effects of age, sex, and residential area

**Fig 1:**
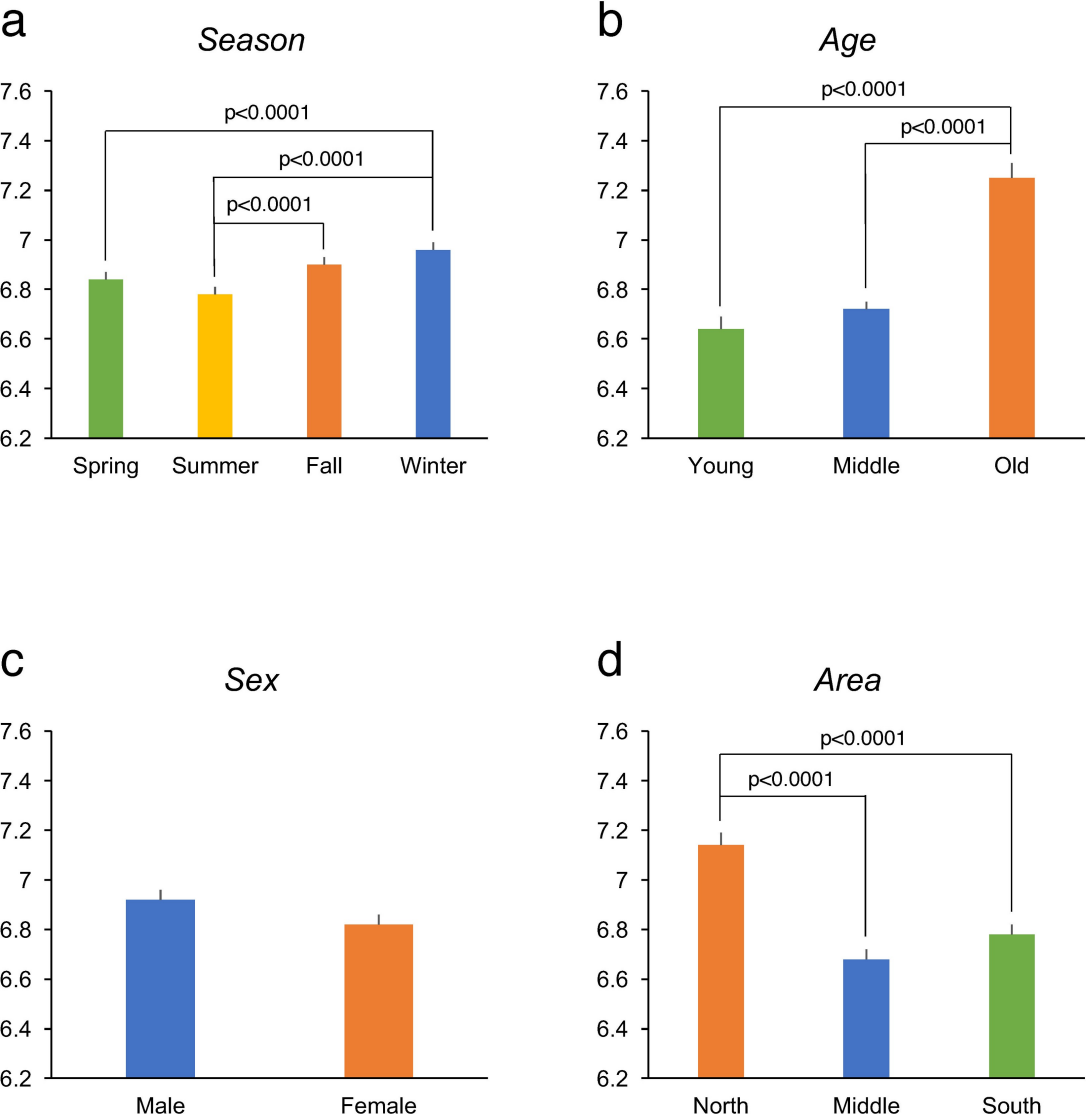
**Sleep duration by season (a), age (b), sex (c), and area (d).** Data are presented as estimated marginal means with standard errors. P-values (Tukey–Kramer) are shown for significant differences.

Table 2 shows a summary of the final linear mixed model using the three-group categorization for age (young, middle, and old). The heterogeneous unstructured covariance model was selected using the likelihood ratio test (P<0.0001). For any season, the old age group had the largest variance, and the middle age group had the lowest variance. A significant main effect was found for *season*, indicating that sleep duration was seasonally changed in our samples. The estimated marginal means of sleep duration for each season in the total sample is shown in Table 3 and illustrated in Fig 1A. Sleep duration was longest in winter and shortest in summer. The difference between the marginal means with standard errors for winter and summer was 0.19±0.03 h (t = 6.43, p<0.0001).

**Table 2:** Summary of the linear mixed model.

Effect	Numerator df	Denominator df	F	p
***Season***	3	1,263	19.04	<0.0001
***Age***	2	664	37.29	<0.0001
***Sex***	1	1,319	12.39	<0.001
***Area***	2	1,330	26.96	<0.0001
***Season* × *age***	6	773	3.49	0.002
***Season* × *sex***	3	1,294	3.31	0.019
***Season* × *area***	6	1,717	2.45	0.023
***Sex* × *age***	2	666	16.62	<0.0001

**Table 3:** Sleep duration for each season by age group.

	Estimated marginal means±SE (h)			
	Spring	Summer	Fall	Winter
**Total (n = 1,388)**	6.84±0.03	6.78±0.03	6.90±0.03	6.96±0.03
**Young (n = 339)**	6.60±0.06	6.64±0.06	6.65±0.06	6.67±0.06
**Middle (n = 709)**	6.69±0.04	6.62±0.04	6.76±0.04	6.79±0.04
**Old (n = 340)**	7.21±0.06	7.06±0.06	7.30±0.07	7.42±0.07

Abbreviations: SE = standard errors.

The model also showed the main effects for *age*, *sex*, and *area* (Fig 1B–1D). Sleep duration in the old age group was longer than that in both the middle and young age groups. Men slept longer than women, with a difference of 6.0 min (p = 0.072, 95% confidence interval: –0.5 to 12.6). Sleep duration was longer in the participants in the north than in those in both the middle and south areas.

**Fig 2:**
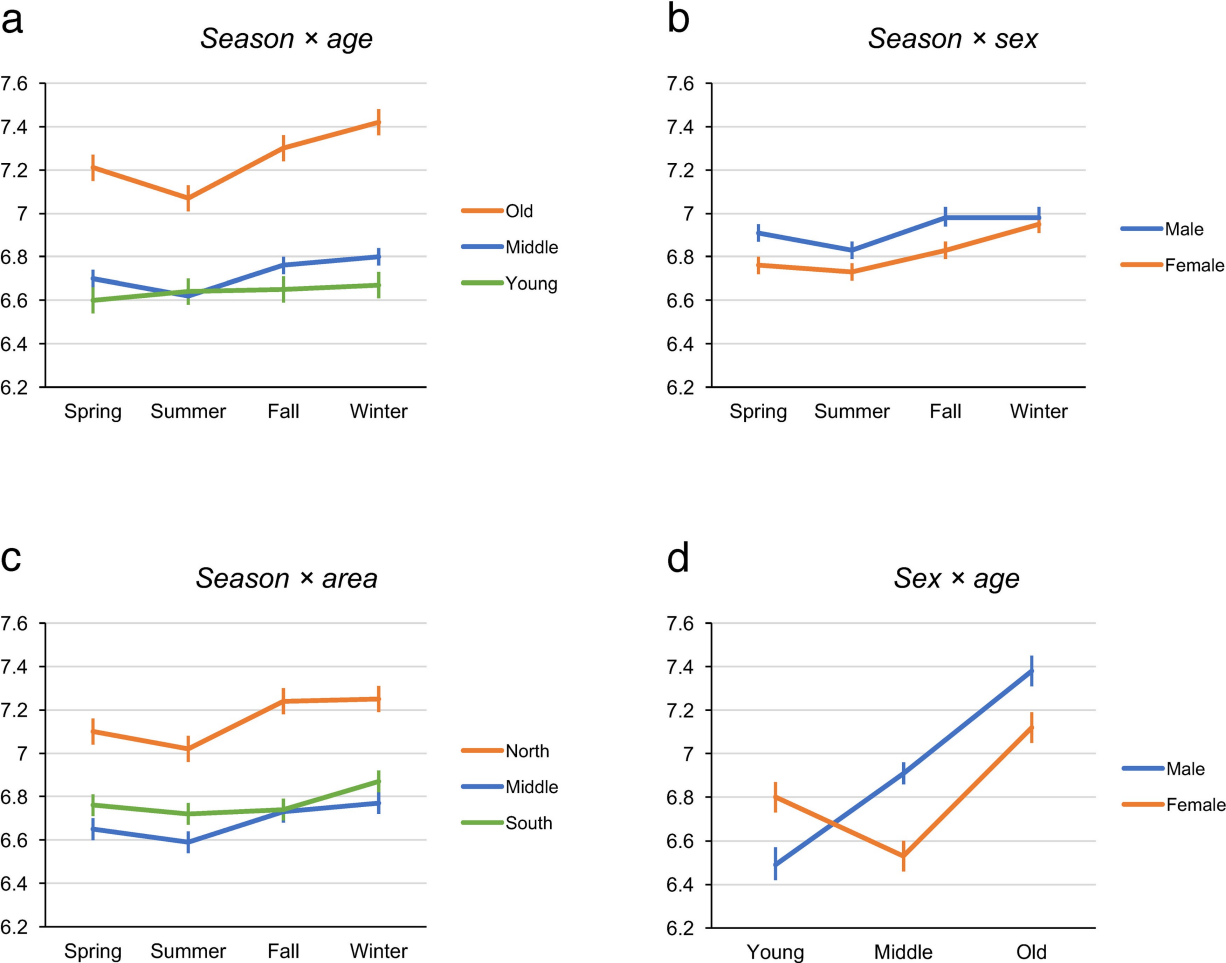
**The interactions found in the model: *season* × *age* (a), *season* × *sex* (b), *season* × *area* (c), and *sex* × *age* (d).** The vertical axis shows the sleep duration (hours). Data are presented as estimated marginal means with standard errors.

The model also showed significant interactions of *season* × *age*, *season* × *sex*, and *season* × *area*, indicating that seasonal changes in sleep duration were influenced by these factors. The estimated marginal means of sleep duration for each season by age group are shown in Table 3 and illustrated in Fig 2A. Sleep duration in the old age group was longer than that in both the young and middle age groups for every season (all p values <0.0001). No significant difference was found for sleep duration between the young and the middle age groups in any season. The participants in the old age group slept longer in winter than in spring (p<0.001) and summer (p<0.0001), and in fall than in summer (p<0.001). Similarly, the participants in the middle age group slept longer in winter than in spring (p = 0.038) and summer (p<0.0001), and in fall than in summer (p<0.001). No significant differences in sleep duration among seasons were found in the young age group, meaning that their sleep duration was not seasonally changed. The differences between the marginal means for winter and summer in the old and middle age groups were 0.36±0.05 h and 0.17±0.03 h, respectively.

The estimated marginal means of sleep duration for each season by sex are plotted in Fig 2B. Men slept shorter in summer than in fall (p = 0.001) and winter (p = 0.003). Women slept longer in winter than in spring (p<0.0001), summer (p<0.0001), and fall (p = 0.004), and in fall than in summer (p = 0.0497). The differences between the marginal means for winter and summer in men and women were 0.15±0.04 h and 0.22±0.04 h, respectively.

The estimated marginal means of sleep duration for each season by area are plotted in Fig 2C. The participants living in the north area slept shorter in summer than in fall (p<0.001) and winter (p<0.001). The participants living in the middle area slept shorter in summer than in fall (p = 0.015) and winter (p<0.001), and in spring than in winter (p = 0.043). The participants living in the south area slept longer in winter than in summer (p = 0.032) and fall (p = 0.031). The differences between the marginal means for winter and summer in the north, middle, and south areas were 0.23±0.05 h, 0.19±0.04 h, and 0.14±0.04 h, respectively.

We also found a significant interaction of *sex* × *age*. The estimated marginal means of sleep duration for each age group by sex are plotted in Fig 2D. In men, sleep duration increased according to age (all group differences were significant). In women, sleep duration was shortest in the middle age group and longest in the old age group (all group differences were significant).

### Seasonal changes in the frequency of DIS, DMS/EMA, and EDS symptoms

Table 4 shows the mean ranks of DIS, DMS/EMA, and EDS symptoms for each season in the total sample and by age group. The Friedman test revealed that the frequencies of DIS (χ^2^ = 8.87, df = 3, p = 0.031), DMS/EMA (χ^2^ = 29.82, df = 3, p<0.0001), and EDS (χ^2^ = 22.41, df = 3, p = 0.006) symptoms changed across seasons in the total sample.

**Table 4:** Mean ranks of DIS, DMS/EMA, and EDS symptoms for each season by age group.

	Mean rank					
	Spring	Summer	Fall	Winter	χ^2^	p
**Total**						
DIS	2.51	2.54	2.47	2.48	8.9	0.031
DMS/EMA	2.58	2.54	2.43	2.45	29.8	<0.0001
EDS	2.56	2.52	2.46	2.46	22.4	<0.0001
**Young**						
DIS	2.55	2.55	2.49	2.42	6.6	NS
DMS/EMA	2.61	2.58	2.47	2.34	25.8	<0.0001
EDS	2.58	2.56	2.42	2.44	12.6	0.006
**Middle**						
DIS	2.52	2.56	2.46	2.47	8.7	0.034
DMS/EMA	2.57	2.56	2.41	2.46	18.3	<0.0001
EDS	2.55	2.50	2.47	2.48	6.7	NS
**Old**						
DIS	2.48	2.50	2.47	2.56	2.6	NS
DMS/EMA	2.56	2.47	2.43	2.54	4.1	NS
EDS	2.55	2.52	2.46	2.47	5.9	NS

Abbreviations: DIS = difficulty initiating sleep, DMS/EMA = difficulty maintaining sleep/early morning awakening, EDS = excessive daytime sleepiness, NS = not significant.

When analyzed according to age group, seasonal changes were found in the frequencies of DMS/EMA (χ^2^ = 25.78, df = 3, p<0.0001) and EDS (χ^2^ = 12.58, df = 3, p = 0.006) symptoms in the young age group, and DIS (χ^2^ = 8.70, df = 3, p = 0.034) and DMS/EMA (χ^2^ = 18.28, df = 3, p<0.0001) symptoms in the middle age group. By contrast, no seasonal changes in the frequencies of DIS, DMS/EMA, or EDS symptoms were found in the old age group.

### Prevalence of DIS, DMS/EMA, and EDS

Table 5 shows the prevalences of DIS, DMS/EMA, and EDS for each season in the total sample. A significant seasonal difference was found for the prevalence of DMS/EMA (P<0.0001), but not for the prevalence of DIS or EDS. Post hoc analysis revealed that the prevalence of DMS/EMA was higher in spring than in fall (p<0.0001) and winter (p<0.0001), and in summer than in fall (p = 0.017) and winter (p = 0.048).

**Table 5:** Prevalence of DIS, DMS/EMA, and EDS for each season in the total sample.

	%			
	Spring	Summer	Fall	Winter
**DIS**	5.48	5.04	4.47	4.54
**DMS/EMA**	10.30	8.93	6.41	6.70
**EDS**	7.28	6.70	5.84	6.05

Abbreviations: DIS = difficulty initiating sleep, DMS/EMA = difficulty maintaining sleep/early morning awakening, EDS = excessive daytime sleepiness.

### Subgroup analysis in the young age group

Of the young age group participants, 88 were categorized as adolescents (15–20 years) and 251 as young adults (21–39 years). Table 6 shows a summary of the final linear mixed model using the four-group categorization for age (adolescents, young adults, middle, and old). The heterogeneous unstructured covariance model was selected using the likelihood ratio test (P<0.0001). Table 7 shows the estimated marginal means of sleep duration for each season in the adolescents and young adults. Sleep duration was not significantly different between the two subgroups for any season. Consistent with the results in the young age group, no significant differences in sleep duration among seasons were found in any of the subgroups.

**Table 6:** Summary of the linear mixed model generated for the subgroup analysis.

Effect	Numerator df	Denominator df	F	p
***Season***	3	1,257	19.10	<0.0001
***Age***	3	328	24.89	<0.0001
***Sex***	1	1,315	12.47	<0.001
***Area***	2	1,328	26.83	<0.0001
***Season* × *age***	9	415	3.84	<0.001
***Season* × *sex***	3	1,295	3.23	0.022
***Season* × *area***	6	1,720	2.34	0.029
***Sex* × *age***	3	327	11.59	<0.0001

**Table 7:** Sleep duration for each season in the adolescents and young adults.

	Estimated marginal means±SE (h)			
	Spring	Summer	Fall	Winter
**Adolescents (n = 88)**	6.54±0.12	6.89±0.13	6.64±0.12	6.68±0.12
**Young adults (n = 251)**	6.63±0.07	6.57±0.07	6.66±0.07	6.67±0.07

Abbreviations: SE = standard error.

Table 8 shows the mean ranks of DIS, DMS/EMA, and EDS symptoms for each time point in the adolescents and young adults. Seasonal changes were found in the frequencies of DMS/EMA (χ^2^ = 27.43, df = 3, p<0.0001) and EDS (χ^2^ = 16.61, df = 3, p = 0.001) symptoms in the young adults; this finding was consistent with the results of the young age group. However, no seasonal changes were observed for any symptoms of sleep problems in the adolescents.

**Table 8:** Mean ranks of DIS, DMS/EMA, and EDS symptoms for each season in the adolescents and young adults.

	Mean rank					
	Spring	Summer	Fall	Winter	χ^2^	p
**Adolescents**						
DIS	2.55	2.56	2.53	2.36	4.3	NS
DMS/EMA	2.55	2.52	2.49	2.45	0.9	NS
EDS	2.66	2.41	2.50	2.43	4.2	NS
**Young adults**						
DIS	2.55	2.55	2.47	2.44	3.7	NS
DMS/EMA	2.64	2.60	2.47	2.30	27.4	<0.0001
EDS	2.55	2.61	2.40	2.44	16.6	0.001

Abbreviations: DIS = difficulty initiating sleep, DMS/EMA = difficulty maintaining sleep/early morning awakening, EDS = excessive daytime sleepiness, NS = not significant.

Table 9 shows the prevalences of DIS, DMS/EMA, and EDS for each season in the young age group. A significant seasonal difference was found for the prevalence of DMS/EMA (p = 0.001). Post hoc analysis revealed that the prevalence of DMS/EMA was higher in spring than in fall (p = 0.039) and winter (p = 0.002), and in summer than in winter (p = 0.019). Although Cochran's Q test revealed a significant seasonal difference for the prevalence of EDS (p = 0.047), no significant differences were found in the post hoc tests. No significant seasonal difference was found for the prevalence of DIS.

**Table 9:** Prevalence of DIS, DMS/EMA, and EDS for each season in the young age group.

	%			
	Spring	Summer	Fall	Winter
DIS	5.90	4.72	3.54	3.24
DMS/EMA	6.78	5.90	3.24	2.06
EDS	15.04	12.68	10.32	10.03

Abbreviations: DIS = difficulty initiating sleep, DMS/EMA = difficulty maintaining sleep/early morning awakening, EDS = excessive daytime sleepiness.

## Discussion

In the present study, we documented seasonal changes in sleep duration and sleep problems by prospectively assessing data collected at four time points (spring, summer, fall, and winter) in the general Japanese population. To our knowledge, this is the first prospective study on the seasonality of sleep duration and sleep problems in a general population living in a temperate region.

The major findings of this study were as follows: 1) in the total sample, seasonal changes were found in sleep duration, with the longest in winter and the shortest in summer; 2) the seasonality of sleep duration was influenced by age, sex, and residential area; 3) in terms of age group, seasonal changes in sleep duration were found in the middle and old age groups, but not in the young age group; 4) in the total sample, seasonal changes were found in the frequencies of DIS, DMS/EMA, and EDS symptoms; and 5) when analyzed according to age group, seasonal changes were found in the frequencies of DMS/EMA and EDS symptoms in the young age group, and DIS and DMS/EMA symptoms in the middle age group; however, no such changes were observed in the old age group.

### The seasonality of sleep duration

We confirmed the findings of previous studies that sleep duration was longer in winter than in summer [12–14, 30] in a general population living in a temperate zone. However, the degree of the winter–summer difference in sleep duration was less than that reported in previous studies. The mean difference in our sample was 11.4 min, while that in previous studies was 24.6–36.0 min [13, 14]. This difference could be due to numerous factors, including differences in sampling, climate, ethnicity, and culture. Furthermore, in previous studies, the seasonality of sleep duration was evaluated retrospectively using the SPAQ. Therefore, the seasonal differences reported in previous studies may have been confounded by recall bias. The difference of time frame also might have influenced the differences in the findings. The SPAQ employs the meteorological definition of seasons to evaluate seasonality in sleep duration, in which the dates of equinoxes and solstices are used to mark the beginning and end of the seasons (for example, summer runs from June 21 to September 20). On the other hand, our data collection frame was generally later than the meteorologically defined season (for example, data in summer was about the participants’ sleep between July 18 and September 30). This 2-week difference in the midpoint of the time frames should be considered when comparing our results with the findings of the previous studies using the SPAQ.

Although it remains unclear why sleep duration is longer in winter and shorter in summer, based on the finding that photoperiod has a substantial influence on sleep length [31], the seasonal difference of day length is likely to be a main factor contributing to the seasonality of sleep duration.

### The seasonality of sleep problems

In the present study, we found significant seasonal changes in the frequencies of DIS, DMS/EMA, and EDS symptoms in the total sample. With regard to prevalence, a seasonal difference was observed for DMS/EMA, being significantly higher in spring and summer.

Itani and colleagues cross-sectionally surveyed the prevalence of insomnia in winter (February) and summer (August) in the general Japanese population [20]. In their survey, they also analyzed the seasonality of the prevalence of insomnia, but did not find any association between insomnia and seasonality. There are several methodological differences between their study and the present study (e.g., study design, method and timing of the data collection, and definition of sleep problems); therefore, the cause of the discrepant results could not be identified.

Epidemiological studies in Northern European countries have demonstrated that insomnia is more prevalent in winter [15–17], although conflicting results have been reported [32]. The mechanisms of mid-winter insomnia in this region remain unclear, but it has been suggested that mid-winter insomnia is a reflection of phase delay of the sleep–wake rhythm caused by a lack of daylight [33]. Total daylight hours during winter in Japan are much longer than those in Northern Europe because of the lower latitude. This might explain why no sleep problems were prevalent in winter in the present study.

A previous study on the seasonality of sleeping problems in Northern and Central Asia and Alaska reported that hot air temperature was associated with premature awakening and difficulties falling and staying asleep [19]. Summer temperatures in Japan are relatively high (the mean maximum temperature in August in Tokyo is around 30°C), which might explain why the prevalence of DMS/EMA was higher in summer than in fall and winter.

In the present study, DMS/EMA was also prevalent in spring. Spring temperatures in Japan are not very high (the mean maximum temperature in May in Tokyo is around 20°C); therefore, other factors rather than air temperatures are likely associated with the high prevalence of DMS/EMA in spring. In Japan, school and company years start in April, so at that time, many people face life-changing events such as continuing on to higher education, getting a new job, or being transferred to a new workplace. Given that psychological stress is considered a major cause of insomnia, the Japanese social system, in which the fiscal and academic years start in April, might be associated with the high prevalence of DMS/EMA observed in spring. Further studies are needed to investigate this association.

### The effect of age on the seasonality of sleep duration and sleep problems

Previous studies have demonstrated that the daily amount of sleep decreases with aging [22]. However, surprisingly, sleep duration in the old age group was longer than that in the other two age groups at every time point. One possible reason for this discrepant result may be the association with job status. In Japan, the retirement age is set at 65 years by most companies; therefore, there is a high possibility that many participants in the old age group had already retired and were getting as much sleep as necessary. On the other hand, there is a possibility that many participants in the young and middle age groups reduced their sleep time to go to school or work. If our speculation is correct, work and school starting times in Japan should be reconsidered. Further studies are needed to investigate the association between school and work status and lack of sleep.

Seasonal changes in sleep duration were most marked in the old age group. The middle age group showed some seasonality of sleep duration, but the degree was less than that of the old age group. On the other hand, the young age group showed no seasonality of sleep duration. This age group difference might be associated with school or job status. That is, old participants may tend to show natural seasonality of sleep duration because they do not have social factors that restrict their sleep. In the subgroup analysis for the young age group, neither the adolescents nor the young adults showed any seasonal changes in sleep duration, which was in accordance with the results of the young adults.

The young age group showed seasonality in DMS/EMA and EDS, and the middle age group showed seasonality in DIS and DMS/EMA; however, the old age group showed no seasonality in any sleep problems. Furthermore, in the subgroup analysis, adolescents also showed no seasonal change in any sleep problems. Taken together, our results suggest that working-age people are more likely to show seasonality of sleep problems in Japan. Since numerous physical, psychological, and social factors change according to life stage, factors associated with seasonal changes in sleep problems in this generation could not be identified. However, future research should be carried out with a focus on this topic, because it could provide important findings for the development of a prevention program for insomnia in working-age people.

### The effects of sex and area on the seasonality of sleep duration

In the present study, we also found that seasonal changes in sleep duration were influenced by sex and residential area. The difference between the marginal means for winter and summer in men was 9.0 min, while that in women was 13.2 min. To our knowledge, no previous reports have described the effect of gender differences on seasonal changes in sleep duration, but previous epidemiological studies using the SPAQ have reported that women are more likely than men to show the seasonality of mood and behavior [13, 34], and that SAD is more prevalent in women [35]. Since there are many biological and behavioral differences between men and women, it is unclear why seasonal changes in sleep duration are greater in women than in men. Further studies are needed to identify the factors associated with gender differences on the seasonality of sleep.

In terms of area difference, the seasonality of sleep duration was most marked in the north area. Japan is a long island that extends north to south, and our survey areas were located between 40.9°N (Aomori) and 31.9°N (Miyazaki). The differences in day length between the longest and shortest days of the year in Aomori and Miyazaki are 5.85 h and 4.17 h, respectively [36], which are considerable differences. Given that photoperiod has been reported to have a substantial influence on sleep length [31], the area differences in the seasonality of sleep duration could have been caused by differences in the seasonal change of day length between the areas. However, other factors such as weather and customs also could have influenced the area differences in the seasonality of sleep. These factors should be considered in future studies.

### Limitations

Our results must be viewed in light of some methodological limitations. First, the light–dark cycle has a major influence on the timing of sleep and wakefulness, but the timing of the data collection did not optimally correspond to the astronomical seasons of the light–dark cycle. To evaluate the winter–summer difference in sleep accurately, data should be collected around the longest (summer solstice) and shortest days of the year (winter solstice). In the present study, the summer solstice of the surveyed years (June 21) was included in one of the time points, but the winter solstice of the surveyed years (December 21 for 2004 and 2005, December 22 for 2006) was not included in any time point. This might have influenced the findings in that a lack of seasonal differences in sleep duration was found in the young population. Second, we collected data at four time points, which might not have been enough to determine a season with worse sleep. Third, the data on sleep duration and sleep problems were based on self-reporting, which could have biased the findings. Fourth, because of the difficulties associated with keeping a detailed 12-day dietary record, households were not selected randomly; however, the regional bias was reduced because the survey was carried out over a wide area of Japan. Fifth, we did not separately assess DMS and EMA. A previous study reported that these symptoms exhibited different patterns of seasonality [19]. Sixth, sleep duration was not assessed separately for work and free days or for weekends or holidays. The difference between seasons in the amount of working days might have confounded the results, especially for the young age group. Seventh, our sampling unit was households, not individuals; therefore, the independence of the samples may not be guaranteed in the strict sense. Since the information regarding which individuals belonged to which households had not been recorded, we could not take the intra-family correlation into account in the statistical analyses, which might have confounded the results. However, it is commonly thought that the main social factors impacting habitual sleep time are school and work schedules, and that the influence of sleep schedules of one’s family members on their own habitual sleep time is much weaker. Finally, many other factors, including psychiatric diagnoses, physical comorbidities, socioeconomic status, the use of sedatives, and other lifestyle factors, were not assessed. In future studies, the above-mentioned factors should be considered.

## Conclusions

In the present study, seasonal effects on sleep and sleep problem were found in Japanese community residents, but the effects between age groups varied. Furthermore, seasonal changes in sleep duration were found to be influenced by sex and residential area.

## Supporting information

S1 File Original questionnaire (in Japanese). (JPG)

S2 File Translated questionnaire (in English). (PDF)

S3 File Data set. (XLSX)
