# Supplementary figures and images for: Seasonal changes in sleep duration and sleep problems: A prospective study in Japanese community residents
Source: PLoS One. 2019 Apr 18;14(4):e0215345. doi: 10.1371/journal.pone.0215345 (PMC6472875; doi:10.1371/journal.pone.0215345)

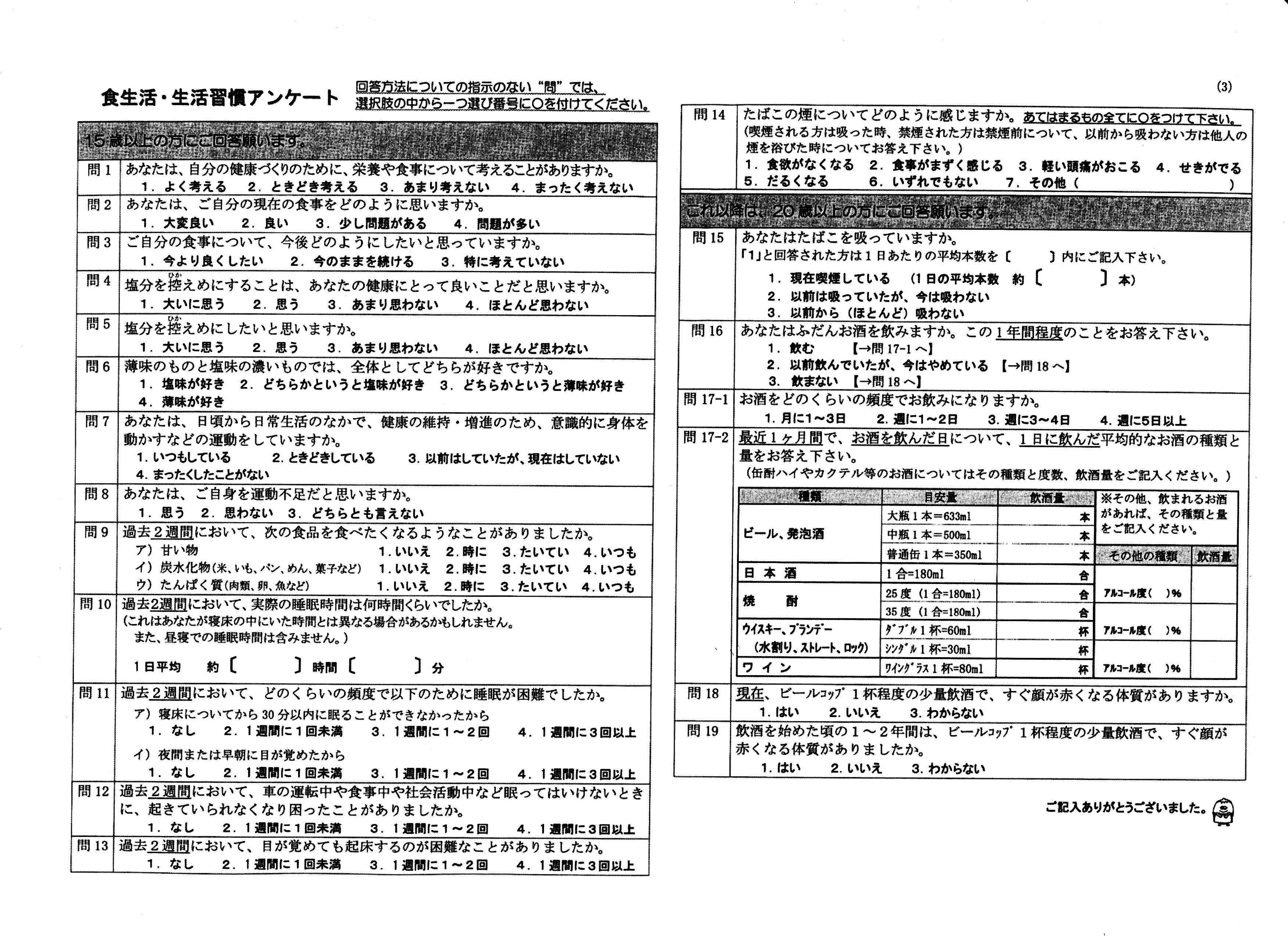

Supplement: S1 File — (JPG) [file pone.0215345.s001.jpg]
