# Supplementary material for: Seasonal changes in sleep duration and sleep problems: A prospective study in Japanese community residents
Source: PLoS One. 2019 Apr 18;14(4):e0215345. doi: 10.1371/journal.pone.0215345 (PMC6472875; doi:10.1371/journal.pone.0215345)
Supplement: S2 File — (PDF) [file pone.0215345.s002.pdf]

Questionnaire regarding eating and lifestyle habits

For questions without specific instructions, please circle one of the numbers that best describes your response.

Please answer the following questions only if you are aged 15 years or older.

Q1: Do you think about nutrition and diet for the purpose of improving your health?

1. Often   2. Sometimes   3. Rarely   4. Not at all

Q2: How would you rate your current diet?

1. Very good   2. Good   3. Somewhat problematic   4. Very problematic

Q3: What would you like to do about your diet in the future?

1. Follow a better diet   2. Maintain my current diet   3. Have not thought about it

Q4: Do you think that reducing your sodium intake is good for your health?

1. Definitely yes   2. Probably yes   3. Probably no   4. Definitely no

Q5: Do you want to reduce your sodium intake?

1. Definitely yes   2. Yes   3. Probably no   4. Mostly no

Q6: Generally speaking, do you prefer more salty or less salty flavoring?

1. Definitely prefer more salty   2. Tend to prefer more salty

3. Tend to prefer less salty   4. Definitely prefer less salty

Q7: In the course of your daily life, do you consciously engage in exercise such as moving your body to maintain and improve your health?

1. Always   2. Sometimes   3. In the past, but not now   4. Never

Q8: Do you feel that you do not engage in enough exercise?

1. Yes   2. No   3. Not sure

Q9: How frequently have you craved the following kinds of food in the past two weeks?

a) Sweets

1. Never   2. Sometimes   3. Often   4. Constantly

b) Carbohydrates (rice, potatoes, bread, noodles, snacks, etc.)

1. Never   2. Sometimes   3. Often   4. Constantly

c) Protein (meat, eggs, fish, etc.)

1. Never 2. Sometimes 3. Often 4. Constantly

Q10: Approximately how many hours of actual sleep per night did you get during the past two weeks? (The time you spent asleep may differ from the time you spent in bed, and does not include naps.)
Approximately (____) hours and (____) minutes per day on average.

Q11: During the past two weeks, how often have you had difficulty sleeping because of the following reasons?
a) I was unable to fall asleep within 30 minutes of getting into bed.
1. Not at all 2. Less than once a week 3. Once or twice a week 4. Three or more times a week

b) I woke up during the night or too early in the morning.
1. Not at all 2. Less than once a week 3. Once or twice a week 4. Three or more times a week

Q12: During the past two weeks, have you ever had trouble staying awake when you were performing a task where you should never fall asleep, such as driving, eating or engaging in social activities?
1. Not at all 2. Less than once a week 3. Once or twice a week 4. Three or more times a week

Q13: In the past two weeks, have you woken up but had difficulty getting out of bed?
1. Never 2. Less than once a week 3. Once or twice a week 4. At least three times a week

Q14: How does tobacco smoke affect you?
Please circle all numbers that apply (current smokers should think about when they smoke, ex-smokers about when they used to smoke, and never-smokers about when they are exposed to second-hand smoke).
1. Ruins my appetite 2. Makes my food taste bad
3. Gives me a light headache 4. Makes me cough
5. Makes me tired 6. None of the above
7. Other - please explain
(____)

Please answer the following questions only if you are aged 20 years or older.

Q15: Have you ever smoked?
If you are a current smoker, please indicate how many cigarettes you smoke per day on average.
1. I currently smoke (____) cigarettes per day on average
2. I used to smoke, but not anymore
3. I have never (or hardly ever) smoked

Q16: Do you drink alcoholic beverages on a regular basis? Please base your answer on your drinking habits in the past year.
1. Yes, I do (skip ahead to Q17-1)

2. I used to drink, but not anymore (skip ahead to Q18)

3. No, I have never drunk alcohol (skip ahead to Q18)

Q17-1: How often do you drink?

1. One to three days a month
2. One to two days a week
3. Three to four days a week
4. Five days a week or more

Q17-2: Please describe what and how much on average you drank on a typical day when consuming alcoholic beverages during the past month.

(For canned *chūhai* or cocktails, please specify the type and alcohol by volume (abv) and the consumed amount.)

Type	Serving size	Consumed amount
Regular and low-malt beer	One large bottle = 633 ml	bottle(s)
	One medium bottle = 500 ml	bottle(s)
	One standard can = 350 ml	bottle(s)
Japanese rice wine	One *go* = 180 ml	*go* (s)
Shōchū	25% abv (1 serving = 180 ml)	serving(s)
	35% abv (1 serving = 180 ml)	serving(s)
Whiskey, brandy (with water, straight, on the rocks)	Double serving = 60 ml	pour(s)
	Single serving = 30 ml	pour(s)
Wine	One glass = 80 ml	glass(es)

* Please describe the types and amounts of any other alcoholic beverages you have consumed.

Type	Consumed amount
abv ( ) %	
abv ( ) %	
abv ( ) %	

Q18: At present, are you physically predisposed to immediate flushing when you consume a small amount of alcohol such as a glass of beer?

1. Yes   2. No   3. Not sure

Q19: During the one or two years after you first started drinking, were you physically predisposed to immediate flushing when you consumed a small amount of alcohol such as a glass of beer?

1. Yes   2. No   3. Not sure

Thank you for completing this questionnaire.
